# Microbiomes of an oyster are shaped by metabolism and environment

**DOI:** 10.1038/s41598-021-00590-2

**Published:** 2021-10-26

**Authors:** Elliot Scanes, Laura M. Parker, Justin R. Seymour, Nachshon Siboni, Michael C. Dove, Wayne A. O’Connor, Pauline M. Ross

**Affiliations:** 1 School of Life and Environmental Sciences, The University of Sydney, Camperdown, NSW 2006 Australia; 2 Climate Change Cluster, University of Technology Sydney, Vicki Sara Building, Ultimo, NSW 2007 Australia; 3 School of Biological, Earth and Environmental Sciences, The University of New South Wales, Kensington, NSW 2052 Australia; 4 New South Wales Department of Primary Industries, Port Stephens Fisheries Institute, Taylors Beach, NSW 2316 Australia

**Keywords:** Climate-change ecology, Ecophysiology, Microbial ecology

## Abstract

Microbiomes can both influence and be influenced by metabolism, but this relationship remains unexplored for invertebrates. We examined the relationship between microbiome and metabolism in response to climate change using oysters as a model marine invertebrate. Oysters form economies and ecosystems across the globe, yet are vulnerable to climate change. Nine genetic lineages of the oyster *Saccostrea glomerata* were exposed to ambient and elevated temperature and PCO_2_ treatments. The metabolic rate (MR) and metabolic by-products of extracellular pH and CO_2_ were measured. The oyster-associated bacterial community in haemolymph was characterised using 16 s rRNA gene sequencing. We found a significant negative relationship between MR and bacterial richness. Bacterial community composition was also significantly influenced by MR, extracellular CO_2_ and extracellular pH. The effects of extracellular CO_2_ depended on genotype, and the effects of extracellular pH depended on CO_2_ and temperature treatments. Changes in MR aligned with a shift in the relative abundance of 152 Amplicon Sequencing Variants (ASVs), with 113 negatively correlated with MR. Some spirochaete ASVs showed positive relationships with MR. We have identified a clear relationship between host metabolism and the microbiome in oysters. Altering this relationship will likely have consequences for the 12 billion USD oyster economy.

## Introduction

Microbiomes are vital to the health and survival of their host^1^. Microbiomes can both influence and be influenced by metabolism in vertebrates such as mammals^2,3^ but this relationship remains unexplored for invertebrate taxa which constitute 97% of all animal species^4^. The metabolism of animals will be remodelled by global climate change as warming accelerates and shifts metabolic requirements. These shifts will be felt most acutely by ectothermic animals, including invertebrates^5^. How invertebrate microbiomes will be affected by this metabolic remodelling and what consequences an altered microbiome may have for their health and survival remains unknown.

Climate change is impacting almost all habitats on Earth. In marine habitats, the oceans are warming, acidifying and de-oxygenating with some evidence of changes in nutrient cycling and primary production affecting marine organisms at multiple trophic levels^6^. Marine invertebrates, unlike vertebrates, possess little control over their internal body condition (e.g. ectothermic, osmoconformers) and rely on metabolic rates to determine the energy available for key functions which govern the production and removal of internal waste such as CO_2_^5^. Metabolic processes of invertebrates will be altered and intensified by climate change, and amongst the most vulnerable invertebrates are the molluscs^7,8^. Farmed molluscs form the foundation of ecosystems and a 29 billion USD economy across the globe, with bivalves such as scallops, mussels and oysters contributing to 90% of mollusc aquaculture^9^. Oyster aquaculture is valued at 12 billion USD^9^ while providing foundational ecosystem services such as three-dimensional habitat and water filtration^10^.

The microbiome of oysters and other bivalves can be altered by ocean warming and acidification^11–13^, with shifts in the microbiome dependent on host genotype^12,14^. Host processes are, therefore, likely to drive changes in the microbiome in response to climate change, yet it remains unknown which host processes are responsible. Metabolic rates are identified as among the most important predictors of resilience to climate change^5^, and previous research has shown that the impact of climate change on metabolism of oysters varies across genotypes, with some being resilient^15^. Oysters capable of maintaining greater growth and extracellular pH have been shown to also possess a greater metabolic rate^16–18^. Metabolic rates may therefore be a key host process shaping the microbiome under climate change. The relationship, however, between microbiome-metabolism and genotype currently remains unstudied in marine molluscs, and it also remains unknown whether warming and acidifying oceans will alter these relationships and impact oyster health and survival^12,16,19^. We examined the relationships of climate change, genotype and microbiome with the Metabolic Rate (MR) and two metabolic by-products (pH_e_ and PCO_2e_) of the Sydney rock oyster, *Saccostrea glomerata*^20^.

## Results and discussion

**Figure 1:**
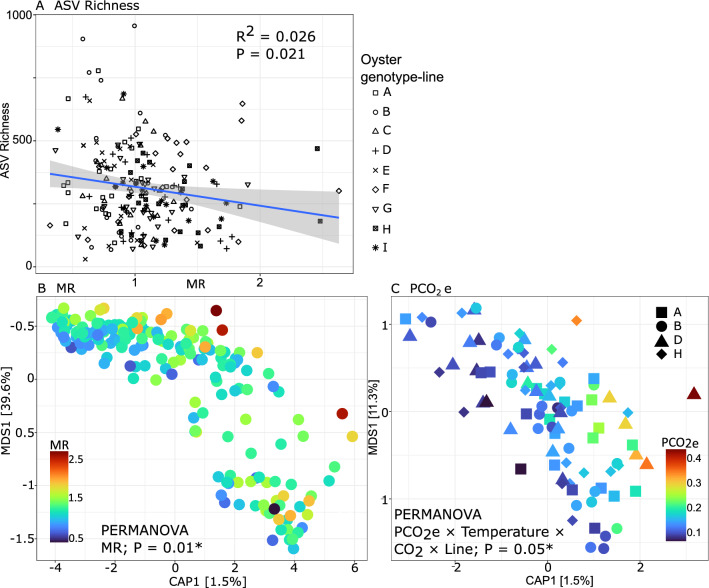
Relationships between microbial communities and physiological variables. (**A**) Relationship between the MR of oysters and ASV Richness. Blue line indicates linear tread (y ~ x) and grey shaded areas indicate 95% confidence intervals. Shapes represent different genotype-lines. Results from linear regression are shown. (**B**) CAP ordination of Weighted unifrac distances calculated from haemolymph oyster-associated bacterial community data. Colours represent MR corresponding to that haemolymph sample. CAP plots were constrained by the physiological variable on the x-axis and were not constrained on the y-axis. Results from PERMANOVA are shown. (**C**) CAP ordination of Weighted unifrac distances calculated from haemolymph oyster-associated bacterial community data. Shapes indicate the four families for which the microbiome was significantly affected by PCO_2e_, colours represent haemolymph PCO_2e_ corresponding to that haemolymph sample. Results from PERMANOVA are shown.

MR, pH_e_ and PCO_2e_ all significantly affected the oyster-associated bacterial community. MR had the strongest relationship with bacterial richness. As MR increased, bacterial richness in oyster haemolymph decreased and was not dependant on genotype-line, temperature or PCO_2_ treatments (Fig. 1A; Table S1). When haemolymph pH_e_ and PCO_2e_ increased, bacterial richness also decreased but this was dependent on genotype, temperature and PCO_2_treatments (ANOVA, Table S1-S3). There was a significant and strong negative correlation between PCO_2e_ and ASV richness in the haemolymph of genotype-line A in the ambient PCO_2_ treatment at 24 °C (PCO_2e_ linear trend = − 10,722.2, *P* < 0.05; Figure S1).

We also found a significant effect of MR as a factor on the community composition of oyster-associated bacteria (PERMANOVA; Table S1). Ordination plots demonstrate partitioning of bacterial communities along a gradient according to oyster MR (Fig. 1B). Haemolymph PCO_2e_ and pH_e_ also affected the oyster-associated bacterial communities, and this effect was again dependent on genotype-line, PCO_2_ and temperature treatments (Tables S2, S3). Haemolymph PCO_2e_ significantly altered oyster-associated bacterial communities in four oyster genotype-lines: A, B D, and H (PERMANOVA, Table S3; Fig. 1C). Similarly, haemolymph pH_e_ significantly altered the community composition of oyster-associated bacteria in only one of the nine oyster genotype-lines (Line F), at 24 °C and ambient PCO_2_ (PERMANOVA; Table S2; Figure S2).

**Figure 2:**
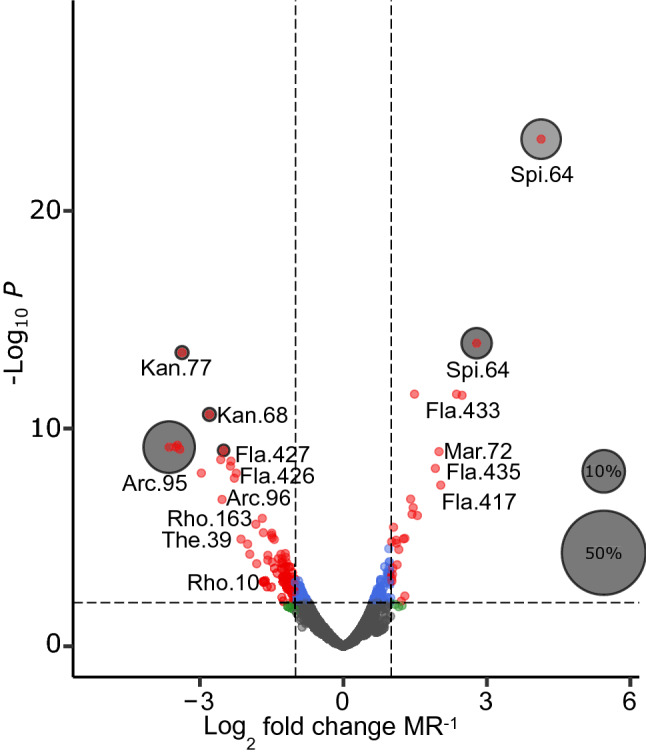
Volcano plot of DESeq2 results showing the ASVs identified to the family level that were significantly affected by MR. Significant *P* values were set at Padj < 0.01. Log2 fold changes are standardised to one unit of change in MR. Red points are those with a Padj < 0.01 and Log2 fold of > 1. Grey circles indicate the relative abundance of ASVs, the top ASVs of interest have been selected.

A total of 152 amplicon sequence variants (ASVs), spanning 43 bacterial families, were significantly affected by MR across all experimental treatments (DESeq GLM analysis, Padj < 0.05; Dataset S1). Of these ASVs, 113 (75% of all affected ASVs and spanning 63% of the differentially abundant bacterial families) were found to be negatively correlated with MR. Three abundant ASVs, one from the *Arcobacteraceae* family, and two from the *Flavobacteriacae* family were found to show the strongest negative correlations with increasing MR (Fig. 2). *Spirochaetaceae* ASVs showed the greatest increases in abundance as MR increased, with one *Spirochaete* ASV increasing by two-fold more than any other ASV.

While this study demonstrated a strong relationship between the haemolymph microbiome of *S. glomerata* and MR, this was not dependent on genotypes or experimental treatments. Effects of PCO_2e_ and pH_e_ were dependent on genotypes, and effects of pH_e_ were also only detected at ambient temperature and PCO_2_. There was no relationship between pH_e_ and the microbiome at elevated temperature and CO_2_. The reasons and consequences for the lack of relationship remain unknown. Our results indicate that MR has an influence on the oyster microbiome independently of the metabolic by-product of CO_2_ and haemolymph pH. One explanation maybe that increased MR produced nitrogenous waste products and may cause shifts in the microbiome. We found that MR increased the abundance of often anaerobic bacteria (e.g. ASVs from the *Thalassospiracae*, *Campylobacterales*, *Desulfovibrio*^21^ and *Anaerovoracaceae*; Dataset S1), which presumably benefit from the low oxygen levels found in the haemolymph of oysters with higher MR. We also found the abundance of *spirochaetes* had a positive relationship with MR (Fig. 2). This is particularly notable given that *spirochaete* ASVs are emerging as a ubiquitous feature of oyster microbiomes^11,22^, yet the nature of the relationship between these bacteria and the oyster host is yet to be determined. Microbiomes play a vital role in the health and survival of their host^1^. While vital, the microbiome also acts as a reservoir for opportunistic pathogens which can cause disease when lowered immune responses or disturbances are beneficial to bacteria^23,24^. Disturbances to the microbiome caused by increased MR as seen in this study, may result in oyster disease and death if the experiment were continued for longer, or conducted in a field environment where there is a greater risk of pathogens and disease.

Metabolites and hormones produced by bacteria are known to regulate the MR and immune functions of humans and animals^2,25^ through mechanisms such as insulin signalling. In this way it is possible that the MR of oysters and subsequently pH_e_ and PCO_2e_, are in fact being shaped by signals from the microbiome.

There are established links between metabolism and the microbiome of humans^2^, with these observations largely limited to vertebrates^3^. Here, we have identified a clear relationship between host metabolism and the composition of the microbiome in an ecologically and economically important marine invertebrate. This relationship was independent of elevated temperature and CO_2_ as well as oyster genotype. Genotypes used in this study have previously shown resilience to the effects of warming and acidification, with elevated MR suggested as a mechanism for this resilience^26^. Overall, these results show that metabolism is likely to be a key host process in driving changes in the microbiome. Changes in oyster metabolism caused by ocean warming and acidification are likely to have consequences for the microbiome^12^. Oysters are vulnerable to new and existing diseases^24^, altering the microbiome-metabolism relationship will likely impact oyster health and survival with consequences for the 12 billion USD oyster economy.

## Methods

More detailed methods can be found in the supplementary material. Data from this experiment on the characterisation of the microbial community and its response to climate change has been previously published in Scanes et al.^12^, therefore, the present study focussed on the interaction of metabolic processes with the microbiome. We examined the links between climate change, metabolism, genotype and microbiome of the Sydney rock oyster, *Saccostrea glomerata*^20^. Nine oyster aquacultural breeding lineages (labelled as genotype-lines A–I) of *S. glomerata*, which are known to differ in their resilience to climate change^12^ were exposed to ambient and elevated temperature and PCO_2_ treatments. All seawater used in acclimation and experimental exposure was collected from Little Beach, Port Stephens (152°9′30.00″E, 32°42′43.03″S), filtered through canister filters to a nominal 5 µm, and stored onsite in 38,000 L polyethylene tanks as a stock of filtered seawater.

Approximately 72 individual *S. glomerata,* from each of the nine families (A-I) were collected from intertidal leases in Cromarty Bay, Port Stephens (152° 4′0.69″E, 32°43′19.69″S). Oysters were held on private leases so a collection permit was not required. Oysters were collected in September 2019 for experiments, meaning all oysters were 22 months old when experiments began. Oysters were placed into a 2000 L fibreglass tank and maintained at 24 °C, a salinity of 35 ppt and ambient PCO_2_ (pH 8.18) for two weeks to acclimate to laboratory conditions. Following acclimation, oysters from each genotype-line were divided among twelve 750 L polyethylene tanks filled with 400 L of filtered seawater (5 µm) at a density of 54 oysters per tank, with each genotype-line represented by six replicate individuals. Treatments consisted of orthogonal combinations of two PCO_2_ concentrations (ambient [400 µatm]; elevated [1000 µatm]) and two temperature treatments (24 and 28 °C). Each combination was replicated across three tanks. Treatments were selected to represent temperatures and PCO_2_ concentrations predicted for 2080–2100 by the IPCC^27^ and reflect measured changes in estuary temperatures reported from south eastern Australia^20^.

Once oysters were transferred to experimental tanks, the PCO_2_ level and temperature were steadily increased in elevated exposure tanks over one week until the experimental treatment level was reached. The elevated CO_2_ level was maintained using a pH negative feedback system (Aqua Medic, Aqacenta Pty Ltd, Kingsgrove, NSW, Australia; accuracy ± 0.01 pH units) bubbling food grade CO_2_ (BOC Australia) through a mixing chamber and into each tank, previously described in^18^. These PCO_2_ levels corresponded to a mean ambient pH_NBS_ of (8.18 ± 0.01) and at elevated CO_2_ levels a mean pH_NBS_ of (7.84 ± 0.01). Temperature was increased and then maintained using 1000 W aquarium heaters in each tank. Oysters were then exposed to their respective treatments for a further four weeks. Oysters were checked daily for mortality; no dead oysters were found in any tanks during the four-week exposure period.

### Haemolymph sampling for DNA extraction

Following exposure to experimental conditions, haemolymph was taken from two replicate oysters, from each genotype-line, from each tank for microbial analysis following the methods previously described in Scanes et al.,^12^. This amounted to six individuals from each genotype-line, in each treatment. Each oyster was opened using an autoclave sterilised shucking knife, ensuring that the pericardial cavity was not ruptured. Excess fluid was tipped off the tissue surface and 200–300 µL of haemolymph was extracted from the pericardial cavity using a new sterile 1 mL needled syringe (Terumo Co.). Samples from two oysters were transferred to two new pre-labelled DNA/RNA free 1 mL tubes (Eppendorf Co.) and immediately frozen at − 80 °C where they were stored until DNA extraction.

We used 16 s rRNA amplicon sequencing to characterise the bacterial microbiome of *S. glomerata* haemolymph following the methods previously described in Scanes et al.^12^. DNA was extracted from 216 oyster haemolymph samples (9 genotype-lines × 4 treatments × 3 replicate tanks × 2 replicate oysters per tank) using the Qiagen DNeasy Blood and Tissue Kit (Qiagen Australia, Chadstone, VIC), according to the manufacturer’s instructions. The bacterial microbiome of the oyster haemolymph was characterised with 16S rRNA amplicon sequencing, using the 341F (CCTACGGGNGGCWGCAG) and 805R (GACTACHVGGGTATCTAATCC) primer pair^28^ targeting the V3-V4 variable regions of the 16S rRNA gene with the following cycling conditions: 95 °C for 3 min, 25 cycles of 95 °C for 30 s, 55 °C for 30 s and 72 °C for 30 s, and a final extension at 72 °C for 5 min. Amplicons were sequenced on the Illumina Miseq platform (2 × 300 bp) following the manufacturer’s guidelines at the Ramaciotti Centre for Genomics, University of New South Wales. Raw data files in FASTQ format were deposited in NCBI Sequence Read Archive (SRA) under Bioproject number PRJNA663356.

### Sequence analysis

Raw demultiplexed data was processed using the Quantitative Insights into Microbial Ecology (QIIME 2 version 2019.1.0) pipeline. Briefly, paired-end sequences were imported (qiime tools import), trimmed and denoised using DADA2 (version 2019.1.0). Sequences were identified at the single nucleotide threshold (Amplicon Sequence Variants; ASV) and taxonomy was assigned using the classify-sklearn QIIME 2 feature classifier against the Silva v138 database^29^. Sequences identified as chloroplasts or mitochondria were also removed. Cleaned data were then rarefied at 6,500 counts per sample.

### Physiological analysis

We measured physiological variables relating to oyster haemolymph metabolic function. These were: extracellular pH (pH_e_), extracellular CO_2_ concentrations (PCO_2e_) and the whole oyster metabolic rate (MR) measured as a standardised rate of oxygen consumption. Physiological measurements were taken from two oysters from each genotype-line in each tank (methods followed that of Parker et al.^16,30^ and Scanes et al.^18^). Oysters were immediately opened without rupturing the pericardial cavity. Haemolymph samples were drawn from the interstitial fluid filling the pericardial cavity chamber of an opened oyster using a sealed 1 mL needled syringe. A 0.2 mL sample was drawn carefully to avoid aeration of the haemolymph. Half of the sample was then immediately transferred to an Eppendorf tube where pH_e_ of the sample was measured at 20 °C using a micro pH probe (Metrohm 827 biotrode). The remaining haemolymph was transferred to a gas analyser (CIBA Corning 965) to determine total CO_2_ (CCO_2_). The micro pH probe was calibrated prior to use with NBS standards at the acclimation temperature and the gas analyser was calibrated using manufacturer guidelines. Two oysters were sampled per genotype-line in each replicate tank. Partial pressure of CO_2_ in haemolymph (PCO_2e_) was calculated from the CCO_2_ using the modified Henderson-Hasselbalch equation according to Heisler^31,32^. Metabolic rate (MR) was determined using a closed respiratory system as previously described in Parker et al.^16^ and Scanes et al.^18^. Briefly, MR was measured in two oysters per genotype-line, per tank by placing oysters in a closed 500 mL glass chamber containing filtered seawater (5 µm) set at the correct treatment conditions. Oxygen concentrations were then measured within the chamber using a fibre optic dipping probe (PreSens dipping probe DP-PSt3, AS1 Ltd, Regensburg, Germany) and recorded (15 s intervals) until the oxygen concentration had been reduced by 20%, the time taken to reduce oxygen by 20% was recorded. Oysters were removed from the chambers, opened and the tissue was dried at 70 °C for 72 h. Tissue was then weighed on an electronic balance (± 0.001 g), and MR was calculated using Eq. (1):

1 $$MR = \frac{{\left[ {V_{r} \times \Delta {\text{C}}_{W} O_{2} } \right]}}{{\Delta t \times {\text{bw}}}}$$

where MR is oxygen consumption normalised to 1 g of dry tissue mass (mg O_2_ g^−1^ dry tissue mass h^−1^), *V*_*r*_ is the volume of the respiratory chamber minus the volume of the oyster (L), ΔC_W_O_2_ is the change in water oxygen concentration measured (mg O_2_L^−1^), Δ*t* is the measuring time (h), bw is the dry tissue mass (g). Equation is modified from Parker et al.^16^.

### Data analysis

It was not possible to measure all variables in each oyster, but rather three individuals were needed to fulfil one replicate set of measurements. PCO_2e_ and pH_e_ could be measured in the same individual however, MR and the microbiome were measured in separate individuals. This meant that measurements were taken from 6 oysters per genotype-line, per replicate tank (each measurement replicated twice). To align physiological data with microbiome data we took a conservative approach where data from PCO_2e_ and pH_e_, MR and the microbiome were randomly matched to individuals from the same genotype-line and replicate tank. This gave us the best approximation and is conservative because it increased variability compared to taking all measurements from the same individual. ANOVA was used to determine the significant (n = 210; *P* < 0.05) effects of factors on bacterial richness. Estimated marginal means of linear trends were used to determine the source of variation when there was a significant interaction between a physiological variable and fixed factor. Normality was checked using the Shapiro–Wilk normality test. To determine the effects of the physiological variables and whether they interacted with our treatments to alter bacterial communities, PERMANOVA (n = 210) using the Adonis procedure were done on Unifrac and Weighted Unifrac with genotype-line (9 levels), PCO_2_ (Ambient and Elevated) and Temperature (24 and 28 °C) as fixed factors, and either PCO_2e_ and pH_e_ or MR as a continuous variable. The homogeneity of dispersion was checked and confirmed for all PERMANOVA. To determine significant differences in the abundance of ASVs dependent on significant physiological variables, the program DESeq2 was used to conduct Generalised Linear Models with a negative binomial distribution and a Benjamini–Hochberg adjusted *P* value to compare abundances of ASVs among treatments^33^. All data analyses downstream of QIIME 2 were done using R v.4.0.1 (R Core team).

## Supplementary Information

Supplementary Information 1.

Supplementary Information 2.

## Data Availability

Raw data files in FASTQ format were deposited in NCBI Sequence Read Archive (SRA) under Bioproject number PRJNA663356.
