# Supplementary material for: Microbiomes of an oyster are shaped by metabolism and environment
Source: Sci Rep. 2021 Oct 26;11:21112. doi: 10.1038/s41598-021-00590-2 (PMC8548560; doi:10.1038/s41598-021-00590-2)
Supplement: Supplementary file 1 — Supplementary Information 1. [file 41598_2021_590_MOESM1_ESM.docx]

**Supplementary information to accompany Scanes et al., 2021 “****Microbiomes of an oyster are shaped by metabolism and environment”**

**SUPPLEMENTARY MATERIALS AND METHODS**

**Experimental animals**

As part of a wider oyster breeding program run by the NSW Department of Primary Industries, genotype-lines of *S. glomerata* genotypes have been selectively bred for three-generations at the Port Stephens Fisheries Institute (PSFI; Dove et al. 2020). The purpose of creating selectively bred lines was to build resilience to disease and fast growth to improve aquaculture profitability, however, previous research has shown that different oyster genotype-lines exhibit dissimilar responses to the effects of climate change (Parker et al. 2011; 2012; 2015; Scanes et al. 2020b, Scanes et al., 2021). Sixty oysters were obtained from each of nine distinct genotype-lines of *S. glomerata.* The genotype-lines used in this study were created at the PSFI in November 2017 using the culture methods of Dove et al. (2020). Each line is created by the pair mating of a male and female oyster from genotype-lines with known pedigree and traits. When juvenile oysters reached a shell length of 1-2 mm they were transferred to purpose-built bags (SEAPA Co. Edwardstown South Australia, 600 x 250 x100 mm) and cultured on intertidal leases in Cromarty Bay, Port Stephens (152° 4'0.69"E, 32°43'19.69"S) where they remained for approximately two years until the beginning of the experiment. The nine genotype-lines from the breeding program were designated: A, B, C, D, E, F, G, H & I, for this study.

**Oyster husbandry, acclimation and experimental exposure**

All seawater used in acclimation and experimental exposure was collected from Little Beach, Port Stephens (152°9'30.00"E, 32°42'43.03"S), filtered through canister filters to a nominal 5 µm, and stored onsite in 38,000 L polyethylene tanks as a stock of filtered seawater (FSW).

Approximately 72 individual *S. glomerata,* from each of the nine genotype-lines (A-I) were collected from intertidal leases in Cromarty Bay, Port Stephens (152° 4'0.69"E, 32°43'19.69"S) in September 2019 for experiments, meaning all oysters were 22 months old when experiments began. Average weight and shell length of experimental oysters was approximately 30 g and 72 mm, respectively. Once collected, oysters were transported to the laboratory at PSFI and gently cleaned of any fouling organisms before being placed into a single 1500 L fibreglass tank containing aerated FSW.

Oysters were maintained in a single 1500 L fibreglass tank for two weeks to acclimate to laboratory conditions at 24 °C and ambient *p*CO_2_ (400 µatm). During all acclimation and experimental exposure, tanks containing oysters received a full water change every second day. This involved removing all oysters from the tank, gently rinsing them with freshwater to remove solid waste and un-eaten food, placing them into a new tank of FSW pre-equilibrated to their *p*CO_2_ and temperature treatment. During all acclimation and experimental exposure, oysters were fed live algae cultured on site comprising of 25 % *Chaetoceros muelleri*, 25 % *Diacronema lutheri*, and 50 % *Tisochrysis lutea* at a rate of 1 x 10^9^ cells/oyster/day.

Following acclimation, oysters from each line were divided among twelve 750 L polyethylene tanks filled with 400 L FSW at a density of 54 oysters per tank, with each line represented by six replicate individuals. Treatments consisted of orthogonal combinations of two *p*CO_2_ concentrations (ambient [400 µatm]; elevated [1000 µatm]) and two temperature treatments (24 and 28 °C). Each combination was replicated across three tanks. Treatments were selected to represent temperatures and *p*CO_2_ concentrations predicted for 2080-2100 by the IPCC (Collins et al. 2013) and reflect measured changes in estuary temperatures reported from south eastern Australia (Scanes et al. 2020b). Oysters remained in experimental treatments for four weeks. Oysters were checked daily for mortality. However, no dead oysters were found in any tanks during the four-week exposure period.

The two *p*CO_2_ levels used in this study (400 µatm, 1000 µatm) were based on the multi-model average projection by the IPCC for 2100 (Collins et al. 2013). These *p*CO_2_ levels corresponded to a mean ambient pH_NBS_ of (8.18± 0.01) and at elevated CO_2_ levels a mean pH_NBS_ of (7.84 ± 0.01). The elevated CO_2_ level was maintained using a pH negative feedback system (Aqua Medic, Aqacenta Pty Ltd, Kingsgrove, NSW, Australia; accuracy ± 0.01 pH units). To determine the pH level corresponding to *p*CO_2_ levels, total alkalinity (TA) was quantified at each water change using triplicate Gran-titration (Gran 1952), and a CO_2_ system calculation program (CO2 SYS; Lewis et al. 1998), using the dissociation constants of (Mehrbach et al. 1973), and the pH level corresponding with the desired *p*CO_2_ level was calculated. Seawater variables including; pH_NBS_, total alkalinity, and salinity were measured each water change, the desired pH values corresponding with *p*CO_2_ levels were then recalculated accordingly. Food grade CO_2_ (BOC Australia) was bubbled directly into independent tanks via a CO_2_ reactor to ensure proper mixing and reduce pH. A pH probe connected to a controlling computer was placed within each tank. Each tank set to elevated *p*CO_2_ was controlled by its own independent pH controlling system. The pH values of each tank were monitored daily and checked daily against another calibrated pH probe (NBS buffers, WTW 3400i).

Once oysters were transferred to experimental tanks, the *p*CO_2_ level and temperature were steadily increased in elevated exposure tanks over one week until the experimental treatment level was reached. Oysters were then exposed to their respective treatments for a further four weeks.

Following exposure to experimental conditions for four weeks, haemolymph was taken from two replicate oysters, from each line, from each tank for microbial analysis. This amounted to six individuals from each Line, in each treatment. To take haemolymph samples, each oyster was opened using an autoclave sterilised shucking knife, ensuring that the pericardial cavity was not ruptured. Excess fluid was tipped off the tissue surface and 200 – 300 µL of haemolymph was extracted from the pericardial cavity using a new sterile 1 mL needled syringe (Terumo Co.). Samples from three oysters were transferred to three new pre-labelled DNA/RNA free 1 mL tubes (Eppendorf Co.) and immediately frozen at -80 °C where they were stored until DNA extraction.

To characterise the bacterial microbiome of *S. glomerata* haemolymph, 16s rRNA amplicon sequencing was used. DNA was extracted from 216 oyster haemolymph samples (9 Genotype-lines × 4 treatments × 3 replicate tanks × 2 replicate oysters per tank) using the Qiagen DNeasy Blood and Tissue Kit (Qiagen Australia, Chadstone, VIC), according to the manufacturer’s instructions. The bacterial microbiome of the oyster haemolymph was characterised with 16S rRNA amplicon sequencing, using the 341F (CCTACGGGNGGCWGCAG) and 805R (GACTACHVGGGTATCTAATCC) primer pair [18] targeting the V3-V4 variable regions of the 16S rRNA gene with the following cycling conditions: 95 °C for 3 min, 25 cycles of 95 °C for 30 s, 55 °C for 30 s and 72 °C for 30 s, and a final extension at 72 °C for 5 min. Amplicons were sequenced on the Illumina Miseq platform (2x300bp) following the manufacturer’s guidelines at the Ramaciotti Centre for Genomics, University of New South Wales. Raw data files in FASTQ format were deposited in NCBI Sequence Read Archive (SRA) under Bioproject number PRJNA663356.

**Sequence analysis**

Raw demultiplexed data was processed using the Quantitative Insights into Microbial Ecology (QIIME 2 version 2019.1.0) pipeline. Briefly, paired-end sequences were imported (qiime tools import), trimmed and denoised using DADA2 (version 2019.1.0), which also removes chimeras (Callahan et al. 2016). Sequences were identified at the single nucleotide threshold (Amplicon Sequence Variants; ASV) and taxonomy was assigned using the classify-sklearn QIIME 2 feature classifier against the Silva v138 database (Quast et al. 2012). Sequences were further cleaned by removing ASVs with reads below 0.005 % ASVs of the summed relative abundance across all 216 samples. Sequences identified as chloroplasts or mitochondria were also removed. Cleaned data were then rarefied at 6,500 counts per sample. Data from QIIME 2 was then exported to R v.4.0.1 (R Core team) for analysis and statistical testing using the packages, “Phyloseq” (McMurdie and Holmes 2013), “DESeq2” (Anders and Huber 2010) and “Vegan” (Dixon, 2003).

**Physiological measurements**

We measured physiological variables relating to oyster haemolymph homeostatic function. These were: extracellular ph (pH_e_), extracellular CO_2_ concentrations (PCO_2e_) and the whole oyster metabolic rate (MR) measured as a standardised rate of oxygen consumption. Physiological measurements were taken from oysters from each line in each tank (methods followed that of Parker et al., 2012; 2018; Scanes et al., 2017). Oysters were immediately opened without rupturing the pericardial cavity. Haemolymph samples were drawn from the interstitial fluid filling the pericardial cavity chamber of an opened oyster using a sealed 1 mL needled syringe. A 0.2 mL sample was drawn carefully to avoid aeration of the haemolymph. Half of the sample was then immediately transferred to an Eppendorf tube where pH_e_ of the sample was measured at 20°C using a micro pH probe (Metrohm 827 biotrode). The remaining haemolymph was transferred to a gas analyser (CIBA Corning 965) to determine total CO_2_ (CCO_2_). The micro pH probe was calibrated prior to use with NBS standards at the acclimation temperature and the gas analyser was calibrated using manufacturer guidelines. Two oysters were sampled per Line in each replicate tank. Partial pressure of CO_2_ in haemolymph (*P*_e_CO_2_) and concentration of [HCO^-^_3_] in the haemolymph ([HCO^-^_3_]_e_) were calculated from the CCO­_2_ using the modified Henderson-Hasselbalch equation (Eqn 1 and 2) according to Heisler (1984, 1986) as found in Riebesell et al. (2010) where molarity of dissolved species = 1.033 M^-1^ L^-1^ (seawater; Hammer et al., 2011), [Na^+^] = 0.55 M (measured previously), and protein concentration of *S. glomerata* = 0.05 g^-1^ L^-1^ (Peters and Raftos, 2003).

$$P_{\text{e}}\text{CO}_{2}=\text{CCO}_{2}\times\left(10^{\text{pH}_{\text{e}}\text{-pK'''}}\times\alpha+\alpha\right)^{-1}$$

**Equation 1.** Where *P*_e_CO_2_ = partial pressure of CO_2_ in haemolymph as calculated (mM), CCO_2_ = total CO_2_ concentration in haemolymph as measured (mM), α = the physical solubility of CO_2_, and pK’’’ is the apparent dissociation constant of carbonic acid in body fluids after Heisler (1986).

$$\left[\text{HCO}^{-}_{3}\right]_{\text{e}}=\text{CCO}_{2}-\left(\alpha\text{CO}_{2}\times P_{\text{e}}\text{CO}_{2}\right)$$

**Equation 2**. Where CCO_2_ = total CO_2_ concentration in haemolymph as measured (mM), αCO_2_ = solubility of CO_2_ in haemolymph calculated after Heisler (1984; 1986; 0.0397 mM^−1^ mmHg^-1^), and *P_e_*CO_2_ = partial pressure of CO_2_ in haemolymph as calculated (mM).

**Metabolic rate and condition index**

Metabolic rate (MR) was determined using a closed respiratory system (Parker et al., 2012) when oysters were immersed. Two individuals were randomly selected from each Line in each replicate tank for measurements. Oysters were placed in individual 500 mL airtight chambers filled with FSW set to the corresponding *p*CO_2_ level of that treatment. Each chamber was fitted with a fibre-optic O_2_ probe (PreSens dipping probe DP-PSt3, AS1 Ltd, Regensburg, Germany). The probes were calibrated using a two-point calibration (0% and 100% air saturated FSW) and all measurements were done at the experimental temperature of 24 or 28°C. The time taken to reduce the percentage oxygen saturation of seawater in the chamber from 100% to 80% was recorded. A “blank” chamber containing only FSW was set up for each treatment to test for bacterial respiration. The change in this chamber over the duration of oyster measurements was negligible and therefore not included in the MR calculation. Time was only recorded when oysters were actively respiring (time that oxygen levels were decreasing). Prior to these MR measurements, feeding had ceased for 24 h to remove any variability associated with digestive metabolism, and individuals were only measured following their allocated immersion time. Following the measurements, oysters were removed from the chambers, opened, and tissue was separated from their shell. Both tissue and shells were dried in an oven at 70˚C for 72 h then weighed using an electronic balance (± 0.001g). MR was calculated for each individual using Eqn 3.

$$\mathrm{MR}=\frac{\left[V_{\mathrm{r}}\times\Delta\mathrm{C}_{\mathrm{W}}\mathrm{O}_{2}\right]}{\Delta t\times\text{bw}}$$

**Equation 3.** Where; MR is oxygen consumption normalised to 1 g of dry tissue mass (mg O_2_ g^-1^ dry tissue mass h^-1^), *V_r_* is the volume of the respiratory chamber minus the volume of the oyster (L), ΔC_W_O_2_ is the change in water oxygen concentration measured (mg O_2_L­^-1^), Δ*t* is the measuring time (h), bw is the dry tissue mass (g) (Parker et al., 2012).

**Data analysis**

It was not possible to measure all variables in each oyster, but rather three individuals were needed to fulfil one replicate set of measurements. PCO_2e_ and pH_e_ could be measured in the same individual however, MR and the microbiome were measured in separate individuals. This meant that measurements were taken from 6 oysters per oyster line, per replicate tank (each measurement replicated twice). To align physiological data with microbiome data we took a conservative approach where data from PCO_2e_ and pH_e_, MR and the microbiome were randomly matched to individuals from the same genotype-line and replicate tank. This gave us the best approximation and is conservative because it increased variability compared to taking all measurements from the same individual.

Richness, Chao1, Unifrac and weighted Unifrac distances were calculated from the rarefied sequence data. To determine the effects of the physiological variables and whether they interacted with our treatments, Richness and Chao1 index were then analysed using linear models with genotype-line (9 levels), *p*CO_2_ (2 levels) and Temperature (2 levels) as fixed factors, and either PCO_2e_ and pH_e_ or MR as a continuous variable. ANOVA was used to determine the significant effects of factors. Estimated marginal means of linear trends were used to determine the source of variation when there was a significant interaction between a physiological variable and fixed factor.

To determine the effects of the physiological variables and whether they interacted with our treatments to alter bacterial communities, PERMANOVA using the Adonis procedure were done on Unifrac and Weighted Unifrac with genotype-line (9 levels), *p*CO_2_ (2 levels) and Temperature (2 levels) as fixed factors, and either PCO_2e_ and pH_e_ or MR as a continuous variable. The homogeneity of dispersion was checked and confirmed using the “β-disper” function (Vegan package; Oksanen et al., 2013) for all factors used in PERMANOVA analyses. To determine significant differences in the abundance of ASVs dependent on significant physiological variables, the “DESeq2” package in R v4.0.1 was used. DESeq2 uses univariate Generalised Linear Models with a negative binomial distribution and a Benjamini-Hochberg adjusted P value to compare abundances of ASVs among treatments (Anders and Huber, 2010). Canonical Analysis of Principal Coordinates (CAP) plots were then created from distance matrices using the “Phyloseq” (McMurdie and Holmes 2013) and “Vegan” (Oksanen et al., 2013) packages. CAP plots were constrained by the physiological variable on the x-axis and were not constrained on the y-axis. ANOVA was used to assess the significance of CAP constraints.

**Supplementary Table 1 (S1).** Analysis of variance table (ANOVA) and Permutational multivariate analysis of variance (PERMANOVA) table created using the Adonis procedure on Weighted Unifrac distances testing the effects of MR (continuous variable) Temperature (fixed – 2 levels), *p*CO_2_ (fixed – 2 levels) and genotype line (Fixed, 9 levels) on the ASV Richness in *S. glomerata* haemolymph.

|  |  | ANOVA |  |  |  | PERMANOVA |  |  |  |
| --- | --- | --- | --- | --- | --- | --- | --- | --- | --- |
|  | Df | Mean Sq | F value | Pr(>F) | Df | MeanSqs | F.Model | R2 | Pr(>F) |
| MR | 1 | 144259 | 5.50 | 0.02* | 1 | 0.55 | 3.40 | 0.02 | 0.01* |
| Temperature | 1 | 426 | 0.02 | 0.90 | 8 | 0.26 | 1.61 | 0.06 | 0.03* |
| CO_2_ | 1 | 8904 | 0.34 | 0.56 | 1 | 0.11 | 0.66 | 0.00 | 0.61 |
| Line | 8 | 25139 | 0.96 | 0.47 | 1 | 0.06 | 0.36 | 0.00 | 0.86 |
| pH_e_ × Temperature | 1 | 25075 | 0.96 | 0.33 | 8 | 0.16 | 0.97 | 0.04 | 0.52 |
| pH_e_ × CO_2_ | 1 | 78108 | 2.98 | `0.09 | 1 | 0.18 | 1.12 | 0.01 | 0.32 |
| Temperature × CO_2_ | 1 | 73875 | 2.82 | 0.10 | 8 | 0.24 | 1.50 | 0.06 | 0.06 |
| pH_e_ × Line | 8 | 40495 | 1.54 | 0.15 | 1 | 0.10 | 0.64 | 0.00 | 0.62 |
| Temperature × Line | 8 | 24354 | 0.93 | 0.50 | 8 | 0.17 | 1.04 | 0.04 | 0.40 |
| CO_2_ × Line | 8 | 28765 | 1.10 | 0.37 | 1 | 0.31 | 1.88 | 0.01 | 0.11 |
| pH_e_ × Temperature × CO_2_ | 1 | 2100 | 0.08 | 0.78 | 8 | 0.16 | 0.97 | 0.04 | 0.52 |
| pH_e_ × Temperature × Line | 8 | 22364 | 0.85 | 0.56 | 8 | 0.10 | 0.61 | 0.02 | 0.95 |
| pH_e_ × CO_2_ × Line | 8 | 41258 | 1.57 | 0.14 | 1 | 0.29 | 1.78 | 0.01 | 0.13 |
| Temperature × CO_2_ × Line | 8 | 22019 | 0.84 | 0.57 | 8 | 0.21 | 1.27 | 0.05 | 0.17 |
| pH_e_ × Temperature × CO_2_ × Line | 8 | 40076 | 1.53 | 0.15 | 8 | 0.16 | 0.99 | 0.04 | 0.47 |
| Residuals | 126 | 26222 |  |  | 126 | 0.16 |  | 0.61 |  |
|  |  |  |  |  | 197 |  |  | 1.00 |  |

**Supplementary Table 2 (S2).** Analysis of variance table (ANOVA) and Permutational multivariate analysis of variance (PERMANOVA) table created using the Adonis procedure on Unifrac distances testing the effects of pH_e_ (continuous variable) Temperature (fixed – 2 levels), *p*CO_2_ (fixed – 2 levels) and genotype-line (Fixed, 9 levels) on the ASV Richness in *S. glomerata* haemolymph.

|  |  | ANOVA |  |  |  | PERMANOVA |  |  |  |  |
| --- | --- | --- | --- | --- | --- | --- | --- | --- | --- | --- |
| Factor | Df | MeanSq | F value | Pr(>F) | Df | MeanSqs | F.Model | R2 | Pr(>F) |  |
| pH_e_ | 1 | 19061 | 0.72 | 0.399 | 1 | 0.20 | 0.94 | 0.00 | 0.54 |  |
| Temperature | 1 | 5748 | 0.22 | 0.643 | 8 | 0.28 | 1.30 | 0.05 | 0.00** |  |
| CO_2_ | 1 | 45756 | 1.72 | 0.192 | 1 | 0.35 | 1.65 | 0.01 | 0.01** |  |
| Line | 8 | 39936 | 1.50 | 0.163 | 1 | 0.26 | 1.21 | 0.01 | 0.14 |  |
| pH_e_ × Temperature | 1 | 37520 | 1.41 | 0.238 | 8 | 0.26 | 1.22 | 0.04 | 0.00** |  |
| pH_e_ × CO_2_ | 1 | 0 | 0.00 | 0.999 | 1 | 0.18 | 0.85 | 0.00 | 0.76 |  |
| Temperature × CO_2_ | 1 | 15711 | 0.59 | 0.444 | 8 | 0.27 | 1.28 | 0.05 | 0.00** |  |
| pH_e_ × Line | 8 | 35216 | 1.32 | 0.238 | 1 | 0.24 | 1.15 | 0.01 | 0.20 |  |
| Temperature × Line | 8 | 20159 | 0.76 | 0.642 | 8 | 0.22 | 1.04 | 0.04 | 0.28 |  |
| CO_2_ × Line | 8 | 55253 | 2.07 | 0.042* | 1 | 0.22 | 1.03 | 0.00 | 0.39 |  |
| pH_e_ × Temperature × CO_2_ | 1 | 3309 | 0.12 | 0.725 | 8 | 0.21 | 0.98 | 0.04 | 0.61 |  |
| pH_e_ × Temperature × Line | 8 | 22336 | 0.84 | 0.571 | 8 | 0.22 | 1.03 | 0.04 | 0.34 |  |
| pH_e_ × CO_2_ × Line | 8 | 15636 | 0.59 | 0.788 | 1 | 0.25 | 1.15 | 0.01 | 0.21 |  |
| Temperature × CO_2_ × Line | 8 | 16649 | 0.62 | 0.756 | 8 | 0.22 | 1.03 | 0.04 | 0.36 |  |
| pH_e_ × Temperature × CO_2_ × Line | 8 | 55889 | 2.10 | 0.04* | 8 | 0.28 | 1.32 | 0.05 | 0.001*** |  |
| Residuals | 138 | 26658 |  |  | 138 | 0.21 |  | 0.63 |  |  |
|  |  |  |  |  | 209 |  |  | 1.00 |  |  |

**Supplementary Table 3 (S3).** Analysis of variance (ANOVA) table and Permutational multivariate analysis of variance (PERMANOVA) table created using the Adonis procedure on Unifrac distances to test the effects of PCO_2e_ (continuous variable) Temperature (fixed – 2 levels), *p*CO_2_ (fixed – 2 levels) and genotype-line (Fixed, 9 levels) on the ASV Richness in *S. glomerata* haemolymph.

|  |  | ANOVA |  |  |  | PERMANOVA |  |  |  |  |
| --- | --- | --- | --- | --- | --- | --- | --- | --- | --- | --- |
|  | Df | Mean Sq | F value | Pr(>F) | Df | MeanSqs | F.Model | R2 | Pr(>F) |  |
| PCO_2e_ | 1 | 20983 | 0.78 | 0.38 | 1 | 0.27 | 1.27 | 0.01 | 0.10 |  |
| Temperature | 1 | 1207 | 0.04 | 0.83 | 8 | 0.27 | 1.27 | 0.05 | 0.00** |  |
| CO_2_ | 1 | 40355 | 1.49 | 0.22 | 1 | 0.35 | 1.63 | 0.01 | 0.01* |  |
| Line | 8 | 38742 | 1.43 | 0.19 | 1 | 0.24 | 1.10 | 0.01 | 0.27 |  |
| PCO_2e_ × Temperature | 1 | 23307 | 0.86 | 0.35 | 8 | 0.22 | 1.03 | 0.04 | 0.30 |  |
| PCO_2e_ × CO_2_ | 1 | 30330 | 1.12 | 0.29 | 1 | 0.28 | 1.29 | 0.01 | 0.08 |  |
| Temperature × CO_2_ | 1 | 3650 | 0.14 | 0.71 | 8 | 0.27 | 1.28 | 0.05 | 0.00** |  |
| PCO_2e_ × Line | 8 | 22988 | 0.85 | 0.56 | 1 | 0.27 | 1.26 | 0.01 | 0.12 |  |
| Temperature × Line | 8 | 14563 | 0.54 | 0.83 | 8 | 0.19 | 0.90 | 0.03 | 0.91 |  |
| CO_2_ × Line | 8 | 51466 | 1.90 | 0.06415 | 1 | 0.23 | 1.08 | 0.01 | 0.28 |  |
| PCO_2e_ × Temperature × CO_2_ | 1 | 67459 | 2.50 | 0.12 | 8 | 0.22 | 1.01 | 0.04 | 0.44 |  |
| PCO_2e_ × Temperature × Line | 8 | 20733 | 0.77 | 0.63 | 8 | 0.25 | 1.15 | 0.04 | 0.04* |  |
| PCO_2e_ × CO_2_ × Line | 8 | 22460 | 0.83 | 0.58 | 1 | 0.26 | 1.19 | 0.01 | 0.16 |  |
| Temperature × CO_2_ × Line | 8 | 25808 | 0.96 | 0.47 | 8 | 0.23 | 1.07 | 0.04 | 0.19 |  |
| PCO_2e_ × Temperature × CO_2_ × Line | 8 | 58382 | 2.16 | 0.03433 * | 8 | 0.25 | 1.14 | 0.04 | 0.05* |  |
| Residuals | 135 | 27023 |  |  | 135 | 0.21 |  | 0.63 |  |  |
|  |  |  |  |  | 206 |  |  | 1 |  |  |


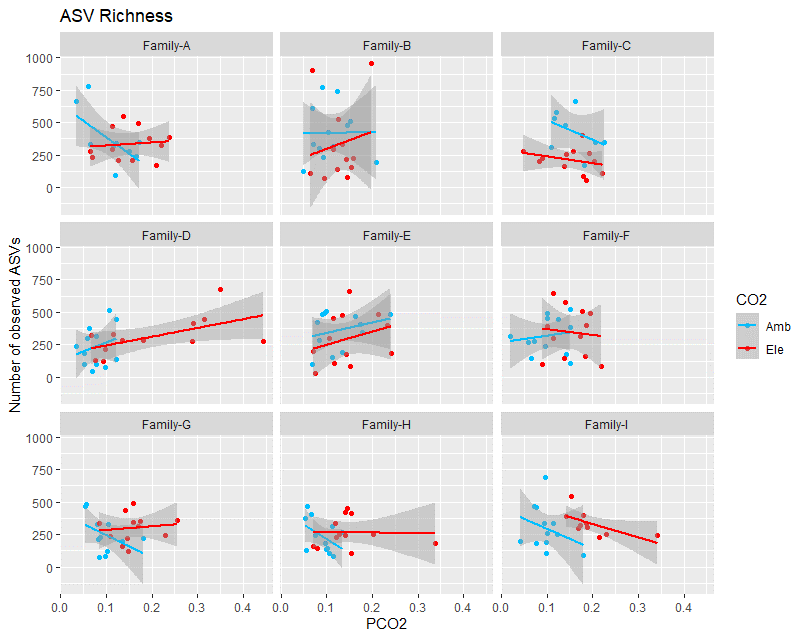


**Supplementary Figure 1 (S1).** Scatter plots of the relationship between PCO_2e_ and ASV richness for each of the nine genotype-lines. Blue data indicate samples from ambient (400µatm) and red indicate elevated (1000µatm) *p*CO_2_ treatments. Only the relationship for Line A, ambient *p*CO_2_ (blue) was found to be significant (Linear trends analysis *P* <0.05). Solid lines indicate linear tread (y ~ x) and grey shaded areas indicate 95 % confidence intervals.


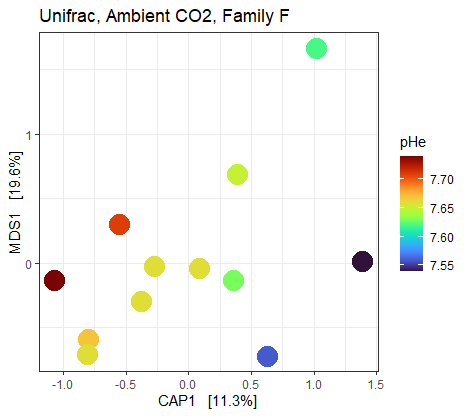


**Supplementary Figure 2 (S2).** CAP ordination of unifrac distances calculated from haemolymph bacterial microbiome data of genotype-line F, at 24°C. Colours represent pH_e_ corresponding to that haemolymph sample. CAP plots were constrained by the physiological variable on the x-axis and were not constrained on the y-axis.

**References cited in this supporting material**

Anders, S. & Huber, W. 2010, 'Differential expression analysis for sequence count data', *Nature Precedings*, pp. 1-.

Collins, M., Knutti, R., Arblaster, J., Dufresne, J.-L., Fichefet, T., Friedlingstein, P., Gao, X., Gutowski, W., Johns, T. & Krinner, G. 2013, 'Long-term climate change: projections, commitments and irreversibility'.

Dove M, Kube P, Lind C *et al.* 2020. *Accelerated Sydney Rock Oyster (SRO) Breeding Research*. FRDC Project 2016–802 Final Report, Port Stephens Fisheries Institute, Taylors Beach.

Dixon, P. 2003, 'VEGAN, a package of R functions for community ecology', *Journal of Vegetation Science*, vol. 14, no. 6, pp. 927-30.

Gran, G. 1952, 'Determination of the equivalence point in potentiometric titrations. Part II', *The Analyst*, vol. 77, no. 920, pp. 661-71.

Hammer, K.M., Kristiansen, E. & Zachariassen, K.E. 2011, 'Physiological effects of hypercapnia in the deep-sea bivalve< i> Acesta excavata</i>(Fabricius, 1779)(Bivalvia; Limidae)', *Marine environmental research*, vol. 72, no. 3, pp. 135-42.

Heisler, N. 1984, 'Acid-base regulation in fishes', *Fish physiology*, vol. 10, no. Part A, pp. 315-401.

Heisler, N. 1986, 'Comparative aspects of acid-base regulation', *Acid-base regulation in animals*, pp. 397-450.

Lewis, E., Wallace, D. & Allison, L.J. 1998, *Program developed for CO2 system calculations*, Carbon Dioxide Information Analysis Center, managed by Lockheed Martin Energy Research Corporation for the US Department of Energy Tennessee.

McMurdie, P.J. & Holmes, S. 2013, 'phyloseq: an R package for reproducible interactive analysis and graphics of microbiome census data', *PloS one*, vol. 8, no. 4, p. e61217.

Mehrbach, C., Culberson, C., Hawley, J. & Pytkowicz, R. 1973, 'Measurement of the apparent dissociation constants of carbonic acid in seawater at atmospheric pressure', *Limnology and Oceanography*, pp. 897-907.

Parker, L.M., O'Connor, W.A., Byrne, M., Dove, M., Coleman, R.A., Pörtner, H.-O., Scanes, E., Virtue, P., Gibbs, M. & Ross, P.M. 2018, 'Ocean acidification but not warming alters sex determination in the Sydney rock oyster, Saccostrea glomerata', *Proceedings of the Royal Society B: Biological Sciences*, vol. 285, no. 1872, p. 20172869.

Parker, L.M., O’Connor, W.A., Raftos, D.A., Pörtner, H.-O. & Ross, P.M. 2015, 'Persistence of Positive Carryover Effects in the Oyster, Saccostrea glomerata, following Transgenerational Exposure to Ocean Acidification', *PloS one*, vol. 10, no. 7, p. e0132276.

Parker, L.M., Ross, P.M., O'Connor, W.A., Borysko, L., Raftos, D.A. & Pörtner, H.O. 2012, 'Adult exposure influences offspring response to ocean acidification in oysters', *Global Change Biology*, vol. 18, pp. 82-92.

Parker, L.M., Ross, P.M. & O’Connor, W.A. 2011, 'Populations of the Sydney rock oyster, *Saccostrea glomerata*, vary in response to ocean acidification', *Marine biology*, vol. 158, no. 3, pp. 689-97.

Peters, R. & Raftos, D.A. 2003, 'The role of phenoloxidase suppression in QX disease outbreaks among Sydney rock oysters (Saccostrea glomerata)', *Aquaculture*, vol. 223, no. 1, pp. 29-39.

Quast, C., Pruesse, E., Yilmaz, P., Gerken, J., Schweer, T., Yarza, P., Peplies, J. & Glöckner, F.O. 2012, 'The SILVA ribosomal RNA gene database project: improved data processing and web-based tools', *Nucleic acids research*, vol. 41, no. D1, pp. D590-D6.

Riebesell, U., Fabry, V.J., Hansson, L. & Gattuso, J.-P. 2010, *Guide to best practices for ocean acidification research and data reporting*, Publications Office of the European Union Luxembourg.

Scanes, E., Parker, L.M., O'Connor, W.A., Dove, M.C. & Ross, P.M. 2020, 'Heatwaves alter survival of the Sydney rock oyster, Saccostrea glomerata', *Marine pollution bulletin*, vol. 158, p. 111389.

Scanes, E., Parker, L.M., O'Connor, W.A., Stapp, L.S. & Ross, P.M. 2017, 'Intertidal oysters reach their physiological limit in a future high-CO2 world', *Journal of Experimental Biology*, vol. 220, no. 5, pp. 765-74.

Scanes, E., Parker, L.M., Seymour, J.R., Siboni, N., King, W.L., Wegner, K.M., Dove, M.C., O’Connor, W.A. & Ross, P.M. 2021, 'Microbiome response differs among selected lines of Sydney rock oysters to ocean warming and acidification ', *FEMS Microbiology Ecology*, vol. In Press.

Scanes, E., Scanes, P.R. & Ross, P.M. 2020, 'Climate change rapidly warms and acidifies Australian estuaries', *Nature Communications*, vol. 11, no. 1, p. 1803.
